# Medullary Thymic Epithelial Cell Antigen-presentation Assays

**DOI:** 10.21769/BioProtoc.4865

**Published:** 2023-11-05

**Authors:** Alexia Borelli, Cloé Zamit, Magali Irla

**Affiliations:** Aix-Marseille University, CNRS, INSERM, CIML, Centre d’Immunologie de Marseille-Luminy, Marseille, France

**Keywords:** Medullary thymic epithelial cells, CD4^+^ T cells, Antigen-presentation assay, Cell purification, Co-culture

## Abstract

Medullary thymic epithelial cells (mTEC) are bona fide antigen-presenting cells that play a crucial role in the induction of T-cell tolerance. By their unique ability to express a broad range of tissue-restricted self-antigens, mTEC control the clonal deletion (also known as negative selection) of potentially hazardous autoreactive T cells and the generation of Foxp3^+^ regulatory T cells. Here, we describe a protocol to assess major histocompatibility complex (MHC) class II antigen-presentation capacity of mTEC to CD4^+^ T cells. We detail the different steps of thymus enzymatic digestion, immunostaining, cell sorting of mTEC and CD4^+^ T cells, peptide-loading of mTEC, and the co-culture between these two cell types. Finally, we describe the flow cytometry protocol and the subsequent analysis to assess the activation of CD4^+^ T cells. This rapid co-culture assay enables the evaluation of the ability of mTEC to present antigens to CD4^+^ T cells in an antigen-specific context.

Key features

• This protocol builds upon the method used by Lopes et al. (2018 and 2022) and Charaix et al. (2022).

• This protocol requires transgenic mice, such as OTIIx*Rag2*-/- mice and the cognate peptide OVA_323–339_, to assess mTEC antigen presentation to CD4^+^ T cells.

• This requires specific equipment such as a Miltenyi Biotec AutoMACS^®^ Pro Separator, a BD FACSAria^TM^ III cell sorter, and a BD^®^ LSR II flow cytometer.

**Graphical overview**


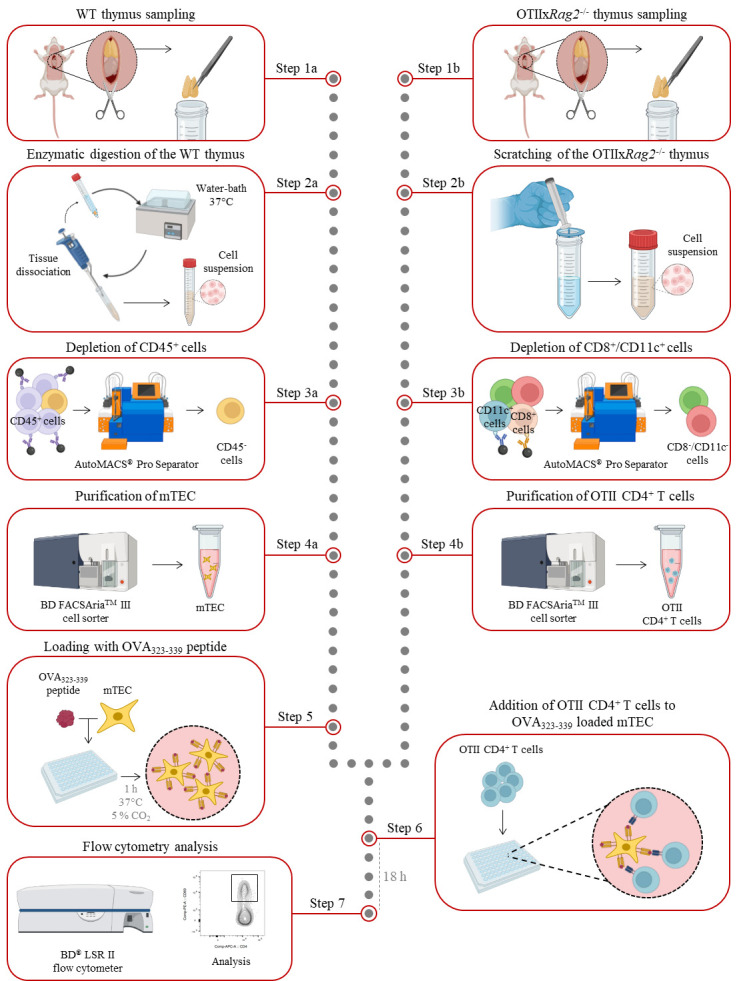


## Background

The thymus is a primary lymphoid organ that ensures the production of functional and self-tolerant naïve T cells. The development of those cells depends on stromal niches composed of thymic epithelial cells (TEC) that provide essential cues for the survival, proliferation, differentiation, and migration of developing T cells, called thymocytes (Irla, 2022). According to their localization in the thymus, TEC are subdivided into two main subsets: cortical (cTEC) and medullary TEC (mTEC). cTEC support early stages of thymopoiesis, including T-cell progenitor homing, T-cell lineage commitment, proliferation and survival of immature thymocytes, and positive selection of CD4^+^ and CD8^+^ single positive thymocytes. In contrast, mTEC control late stages of T-cell development, i.e., the clonal deletion of highly autoreactive thymocytes and the generation of mature Foxp3^+^ regulatory T cells (T_reg_). mTEC are commonly subdivided into two main subsets based on the expression of MHC class II (MHC-II) and CD80 molecules: mTEC^lo^ (MHC-II^lo^CD80^lo^) and mTEC^hi^ (MHC-II^hi^CD80^hi^), with mTEC^lo^ containing progenitors capable of differentiating into mTEC^hi^ (Gray et al., 2006; Gäbler et al., 2007; Miragaia et al., 2018). mTEC constitute a crucial antigen reservoir due to their unique capacity to express a wide range of tissue-restricted self-antigens. The expression of the majority of these tissue-restricted self-antigens is mediated by the transcription factors Aire (Autoimmune regulator) and Fezf2 (Fez family zinc finger 2), which regulate the expression of 3,000–4,000 and 600–700 genes, respectively (Sansom et al., 2014; Takaba et al., 2015). mTEC are bona fide antigen-presenting cells, as they can efficiently induce the clonal deletion of highly autoreactive CD4^+^ and CD8^+^ T cells, as well as the differentiation of CD4^+^ T cells towards the Foxp3^+^ T_reg_ cell lineage (Aschenbrenner et al., 2007; Hinterberger et al., 2010; Aichinger et al., 2013). Interestingly, our lab recently showed that Aire^+^ mTEC also have the ability to restimulate recirculating Foxp3^+^ T_reg_ upon their re-entry in the thymus in an antigen-specific manner (Charaix et al., 2022). It is important to notice that mTEC–CD4^+^ T cell interactions act as bidirectional signals, as not only mTEC are crucial for CD4^+^ T cell selection but also CD4^+^ T cells control mTEC differentiation ( Irla et al., 2008; Lopes et al., 2015 and 2022).

The antigen-presentation capacity of mTEC to CD4^+^ T cells can be assessed by co-culture experiments using variable amounts of mTEC or peptide concentration (Aschenbrenner et al., 2007; Wirnsberger et al., 2009; Hinterberger et al., 2010; Lopes et al., 2018 and 2022; Charaix et al., 2022). This in vitro co-culture protocol describes the steps to analyze the antigen-presentation capacity of mTEC through the activation of CD4^+^ T cells in an antigen-specific manner. It describes the different steps of thymus collection, enzymatic digestion, immunostaining, cell sorting of mTEC and CD4^+^ T cells, peptide-loading of mTEC, and the co-culture between these two cell types. Although this approach may be limited by the fact that it is an in vitro assay, it has the advantage of specifically and rapidly evaluating, within two days, the antigen-presentation capacity of mTEC. This protocol can also be used to assess the antigen-presentation capacity of human mTEC or dendritic cell subsets, the latter also implicated in the induction of T-cell tolerance (Lopes et al., 2015; Irla, 2022).

## Materials and reagents

**Biological species**

1. C57BL/6 WT mice at the age of 4–6 weeks (Charles River)
2. OTIIx*Rag2*-/- mice on a C57BL/6 background at the age of 4–6 weeks (Shinkai, 1992; Barnden et al., 1998)

*Note: Mice are age- and sex-matched.*

**Reagents**

1. Fetal bovine serum (Pan Biotech, catalog number: P30-3306)
2. Bovine serum albumin (Sequens IVD, catalog number: 1000-70)
3. Liberase^TM^ (Roche, catalog number: 5401127001)
4. DNase I (Roche, catalog number: 10104159001)
5. HBSS, without calcium and magnesium, no phenol red (Thermo Fisher Scientific, Gibco^TM^, catalog number: 14175-053)
6. PBS 1×, without calcium and magnesium (Thermo Fisher Scientific, Gibco^TM^, catalog number: 10010-023)
7. EDTA, 0.5 M pH 8.0 (Thermo Fisher Scientific, Invitrogen, catalog number: AM9261)
8. L-glutamine (Thermo Fisher Scientific, Gibco^TM^, catalog number: 25030024)
9. Penicillin and streptomycin (Thermo Fisher Scientific, Gibco^TM^, catalog number: 15140-122)
10. 2-mercaptoethanol (Thermo Fisher Scientific, Gibco^TM^, catalog number: 31350-010)
11. Sodium pyruvate (Thermo Fisher Scientific, Gibco^TM^, catalog number: 11360-039)
12. D-MEM (Thermo Fisher Scientific, Gibco^TM^, catalog number: 41965-039)
13. Red blood cell (RBC) lysis buffer (Thermo Fisher Scientific, catalog number: 00-4333-57)
14. Antibodies:
  - Purified Rat Anti-Mouse CD16/CD32 (Mouse BD Fc Block^TM^) (BD Biosciences, clone 2.4G2, catalog number: 553142)
  - Biotin anti-mouse CD45 antibody (BioLegend, clone 30-F11, catalog number: 103104)
  - Biotin anti-mouse CD8 antibody (BioLegend, clone 53-6.7, catalog number: 100704)
  - Biotin anti-mouse CD11c antibody (BioLegend, clone N418, catalog number: 117304)
  - PE/Cyanine7 conjugated anti-mouse EpCAM (CD326) antibody (BioLegend, clone G8.8, catalog number: 118216)
  - PE conjugated anti-mouse Ly51 (CD249) antibody (BD Biosciences, clone BP-1, catalog number: 553735)
  - Fluorescein conjugated UEA1 (Vector Laboratories, catalog number: FL-1061-5)
  - Brilliant Violent 421^TM^ conjugated anti-mouse CD80 antibody (BioLegend, clone 16-10A1, catalog number: 104726)
  - Alexa Fluor^®^ 700 conjugated anti-mouse MHC-II (I-A/I-E) antibody (BioLegend, clone M5/114.15.2, catalog number: 107622)
  - Brilliant Violent 421^TM^ conjugated anti-mouse CD4 antibody (BD Biosciences, clone RM4.5, catalog number: 740007)
  - PerCP-Cy^TM^5.5 conjugated anti-mouse CD8 antibody (BD Biosciences, clone 53-6.7, catalog number: 551162)
  - PE conjugated anti-mouse CCR6 antibody (BioLegend, clone 29-2L17, catalog number: 129804)
  - PE/Cyanine7 conjugated anti-mouse CD25 antibody (BioLegend, clone PC61, catalog number: 102016)
  - APC conjugated anti-mouse CD69 antibody (BioLegend, clone H1.2F3, catalog number: 104514)
15. Anti-Biotin MicroBeads (Miltenyi Biotec, catalog number: 130-090-485)
16. Ovalbumine_323–339_ (OVA_323–339_) peptide (Polypeptide group SC1303 then Anaspec Inc., catalog number: AS-27024)

**Solutions**

1. Digestion buffer (see Recipes)
2. FACS buffer (see Recipes)
3. AutoMACS buffer (see Recipes)
4. Complete culture medium (see Recipes)
*Note: All buffers and culture medium are filtered through a 0.22 μm filter.*

**Recipes**

1. **Digestion buffer**
  **Table:** 

Reagent	Final concentration	Quantity
HBSS 1×	n/a	9.9 mL
Liberase^TM^	50 μg/mL	100 μL
DNase I	100 μg/mL	10 μL
Total	n/a	10 mL
*Note: Enzymes are sensitive to temperature; therefore, prepare before use.*
2. **FACS buffer**
  **Table:** 

Reagent	Final concentration	Quantity
PBS 1×	n/a	1,980 mL
EDTA (0.5 M, pH 8.0)	5 mM	20 mL
Bovine serum albumin	0.5%	10 g
Total	n/a	2,000 mL
*Note: Store up to one month at 4 °C and keep on ice for the duration of the protocol.*
3. **AutoMACS buffer**
  **Table:** 

Reagent	Final concentration	Quantity
PBS 1×	n/a	1,982 mL
EDTA (0.5 M, pH 8.0)	2 mM	8 mL
Fetal bovine serum	0.5%	10 mL
Total	n/a	2,000 mL
*Note: Store up to one month at 4 °C and keep on ice for the duration of the protocol.*
4. **Complete culture medium**
  **Table:** 

Reagent	Final concentration	Quantity
D-MEM	n/a	435 mL
Fetal bovine serum	10%	50 mL
L-Glutamine Sodium pyruvate 2-mercaptoethanol Penicillin and streptomycin	2 mM 1 mM 2 × 10^-5^ M 100 IU/mL	5 mL 5 mL 500 μL 5 mL
Total	n/a	500 mL
*Note: Store up to one month at 4 °C. One hour before use, place the medium in a water bath at 37 °C.*

**Laboratory supplies**

1. Curved forceps (Fine Science Tools, catalog number: 11271-30)
2. Straight forceps (Fine Science Tools, catalog number: 11064-07)
3. Dissection scissors (Bochem^TM^, catalog number: 4070)
4. Micropipette tips [Sarstedt, catalog number: 70.3050.205 (1,000 μL); Starlab, catalog numbers: S1111-1810 (200 μL) and S1110-3700 (0.1–10 μL)]
5. 5 mL Falcon round-bottom polystyrene tube (Fisher Scientific, Corning, catalog number: 352008)
6. 15 mL tube (Sarstedt, catalog number: 62.554.502)
7. 50 mL tube (Sarstedt, catalog number: 62.547.254)
8. 1.5 mL tubes (Eppendorf, Dutscher, catalog number: 033305)
9. 96-well U-bottom cell culture plate (Cellstar^®^, Greiner bio-one, catalog number: 650180)
10. 96-well V-bottom cell culture plate (Cellstar^®^, Greiner bio-one, catalog number: 651180)
11. 70 μm pore cell strainers (Sarstedt, catalog number: 83.3945.070)
12. 30 μm pre-separation cell strainers (Miltenyi Biotec, catalog number: 130-041-407)
13. 1 mL syringe (Terumo; catalog number: SS+01H1)
14. Steritop E-GP sterile vacuum 0.22 μm filtration system (Millipore Merck, catalog number: SEGPT0045)

## Equipment

1. Micropipettes 0.1–2.5 μL, 2–20 μL, 20–200 μL, and 100–1,000 μL (Eppendorf, Thermo Fisher Scientific, catalog number: 05-403-152)
2. Centrifuge (Eppendorf, Thermo Fisher Scientific, model: 5810R and 5415R)
3. 37 °C 5% CO_2_ incubator Forma Scientific 3548 (Labexchange, Forma Scientific, catalog number: B00032422)
4. 4 °C refrigerator (Candy)
-20 °C freezer (Liebherr)
6. Water bath (Grant Instruments)
7. AutoMACS^®^ Pro Separator (Miltenyi Biotec, catalog number: 130-092-545)
8. BD FACSAria^TM^ III cell sorter (BD Biosciences)
9. BD^®^ LSR II flow cytometer (BD Biosciences)

## Software and datasets

1. FlowJo (BD Biosciences, Version 10.8.1)
2. BD FACSDiva^TM^ software (BD Biosciences, Version 9.0)

## Procedure

- **Thymus withdrawal**
  1. Mouse euthanasia was performed through 100% CO_2_ inhalation at a flow rate of 20 L/min in accordance with National and European laws for laboratory animal welfare (EEC Council Directive 2010/63/UE) and the Marseille Ethical Committee for Animal experimentation no. 14. Verify that the mouse is unresponsive to a toe pinch.
  2. Place the mouse on its back and pin each limp down to the dissection board. Spray with 70% ethanol to sterilize the mouse body.
  3. Open the thoracic cavity with scissors and carefully expose the heart and thymus above without cutting major blood vessels.
  4. Gently remove the thymus with a pair of forceps.
*Note: Make sure to remove fat and connective tissue from the thymus.*
  5. Transfer the thymus into a 15 mL collection tube pre-filled with 2 mL of enzymatic digestion buffer for WT mice or 2 mL of cold FACS buffer for OTIIx*Rag2*-/- mice.
- **Thymic epithelial cell isolation**
  1. Digest the thymus from WT mice in a water bath at 37 °C for 15 min in 2 mL of digestion buffer; then, dissociate the tissue by approximately 20 recurrent aspirations through a 1,000 μL tip.
*Note: Agitate the fractions gently during the digestion process and avoid bubbles.*
  2. Filter thymic cells through a 70 μm pore cell strainer into a 50 mL tube with 3 mL of cold FACS buffer.
  3. Collect remnant undigested tissue from the cell strainer with forceps and return it to the 15 mL tube containing digestion buffer.
  4. Digest remnant tissue in a water bath at 37 °C for 15 min. Then, dissociate the tissue by approximately 20 recurrent aspirations through a 1,000 μL tip.
  5. Filter thymic cells through a 70 μm pore cell strainer into the 50 mL tube with 3 mL of cold FACS buffer.
  6. Repeat steps B3–B5 until complete tissue digestion.
  7. Centrifuge at 450× *g* for 5 min at 4 °C.
  8. Remove the supernatant and resuspend the cell pellet in 1 mL of RBC lysis buffer for 3 min.
  9. Wash the cells by adding 10 mL of cold FACS buffer.
  10. Centrifuge at 450× *g* for 5 min at 4 °C.
  11. Prepare the biotin antibody mix No. 1: dilute the biotin anti-mouse CD45 antibody in cold FACS buffer at the final concentration of 2 μg/mL.
  12. After the centrifugation, remove the supernatant and resuspend the cell pellet with 500 μL of the biotin antibody mix No. 1.
  13. Incubate the cells for 15 min at 4 °C.
  14. Wash the cells by adding 10 mL of cold FACS buffer.
  15. Centrifuge at 450× *g* for 5 min at 4 °C.
  16. Prepare the anti-biotin microbead mix: dilute 50 μL of anti-biotin microbeads in 450 μL of cold FACS buffer.
  17. After the centrifugation, remove the supernatant and resuspend the cell pellet with 500 μL of the anti-biotin microbead mix.
  18. Incubate the cells for 15 min at 4 °C.
  19. Wash the cells by adding 10 mL of cold FACS buffer.
  20. Centrifuge at 450× *g* for 5 min at 4 °C.
  21. Remove the supernatant and resuspend the cell pellet in 4 mL of cold AutoMACS buffer.
  22. Pass the cells through a pre-separation filter into a 15 mL tube to remove cell clumps that may clog the AutoMACS^®^ Pro Separator columns.
  23. Proceed to magnetic separation with the AutoMACS^®^ Pro Separator using the DepleteS program.
  24. Keep the CD45- fraction and discard the CD45^+^ fraction.
  25. Centrifuge the CD45- fraction at 450× *g* for 5 min at 4 °C.
  26. Prepare the antibody mix in cold FACS buffer for mTEC staining with the following concentrations:
    Purified Rat Anti-Mouse CD16/CD32: final concentration 1 μg/million cells.
    PE/Cyanine7 conjugated anti-mouse EpCAM antibody: final concentration 60 ng/mL.
    PE conjugated anti-mouse Ly51 antibody: final concentration 60 ng/mL.
    Fluorescein conjugated UEA1: final concentration 6.25 μg/mL.
*Note: Based on the desired mTEC subset for the co-culture assay, include anti-mouse CD80 and anti-mouse I-A/I-E antibodies to discriminate mTEC^lo^ and mTEC^hi^ with:*
*Brilliant Violent 421^TM^ conjugated anti-mouse CD80 antibody: final concentration 0.5 μg/mL.*
*Alexa Fluor^®^ 700 conjugated anti-mouse I-A/I-E antibody: final concentration 0.9 μg/mL.*
  27. Remove the supernatant and resuspend the cell pellet with 500 μL of antibody mix for mTEC staining.
  28. Incubate the cells for 15 min at 4 °C.
  29. Wash the cells by adding 10 mL of cold FACS buffer.
  30. Centrifuge at 450× *g* for 5 min at 4 °C.
  31. Remove the supernatant and resuspend the stained cell pellet with 500 μL of cold FACS buffer.
  32. Keep on ice until cell sorting.
  33. Sort total mTEC (or alternatively mTEC^lo^ and mTEC^hi^) with a BD FACSAria^TM^ III cell sorter (Figure 1). Cells are collected in 1.5 mL Eppendorf tubes containing 100 μL of complete culture medium.
    **Figure 1.**
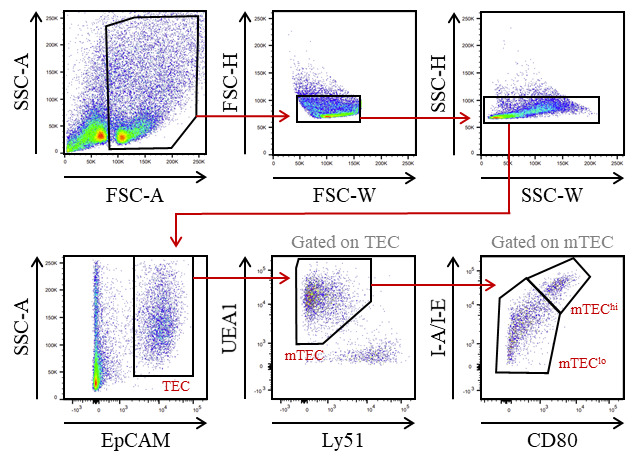
Gating strategy used to purify medullary thymic epithelial cells (mTEC) from WT mice by cell sorter after AutoMACS pre-enrichment of CD45- cells. EpCAM^+^ TEC were divided into mTEC (UEA-1^+^Ly51^lo^ cells) and subsequently into mTEC^lo^ (UEA-1^+^Ly51^lo^CD80-I-A/I-E- cells) and mTEC^hi^ (UEA-1^+^Ly51^lo^CD80^+^ I-A/I-E^+^ cells) based on the level of the CD80 and MHC-II molecules.
- **Isolation of OTII CD4^+^ T cells**
  1. Mechanically dissociate the thymus from OTIIx*Rag2*-/- mice by scratching it in cold FACS buffer on a 70 μm pore cell strainer fixed on a 50 mL tube with the plunger of a 1 mL syringe.
  2. Centrifuge at 450× *g* for 5 min at 4 °C.
  3. Remove the supernatant and resuspend the cell pellet in 1 mL of RBC lysis buffer for 3 min.
  4. Wash the cells by adding 10 mL of cold FACS buffer.
  5. Centrifuge at 450× *g* for 5 min at 4 °C.
  6. Prepare the biotin antibody mix No. 2: Dilute the biotin anti-mouse CD8 and CD11c antibodies in cold FACS buffer at the final concentration of 2.5 μg/mL and 1.5 μg/mL, respectively.
  7. After the centrifugation, remove the supernatant and resuspend the cell pellet with 500 μL of the biotin antibody mix No. 2.
  8. Incubate the cells for 15 min at 4 °C.
  9. Wash the cells by adding 10 mL of cold FACS buffer.
  10. Centrifuge at 450× *g* for 5 min at 4 °C.
  11. Prepare the anti-biotin microbead mix: dilute 50 μL of anti-biotin microbeads in 450 μL of cold FACS buffer.
  12. After the centrifugation, remove the supernatant and resuspend the cell pellet with 500 μL of the anti-biotin microbead mix.
  13. Incubate the cells for 15 min at 4 °C.
  14. Wash the cells by adding 10 mL of cold FACS buffer.
  15. Centrifuge at 450× *g* for 5 min at 4 °C.
  16. Remove the supernatant and resuspend the cell pellet in 4 mL of cold AutoMACS buffer.
  17. Pass the cells through a pre-separation filter into a 15 mL tube to remove cell clumps that may clog the AutoMACS^®^ Pro Separator columns.
  18. Proceed to magnetic separation with the AutoMACS^®^ Pro Separator, using the Deplete program.
  19. Keep the CD8- and CD11c- fraction and discard the other fraction.
  20. Centrifuge the CD8- and CD11c- fraction at 450× *g* for 5 min at 4 °C.
  21. Prepare the antibody mix in cold FACS buffer for CD4^+^ T cells staining with the following concentrations:
    Brilliant Violent 421^TM^ conjugated anti-mouse CD4 antibody: final concentration 1 μg/mL.
    PerCP-Cy^TM^ 5.5 conjugated anti-mouse CD8 antibody: final concentration 1 μg/mL.
    PE conjugated anti-mouse CCR6 antibody: final concentration 0.7 μg/mL.
    PE/Cyanine7 conjugated anti-mouse CD25 antibody: final concentration 0.5 μg/mL.
    APC conjugated anti-mouse CD69 antibody: final concentration 1 μg/mL.
  22. Remove the supernatant and resuspend the cell pellet with 500 μL of antibody mix for CD4^+^ T-cells staining.
  23. Incubate the cells for 15 min at 4 °C.
  24. Wash the cells by adding 10 mL of cold FACS buffer.
  25. Centrifuge at 450× *g* for 5 min at 4 °C.
  26. Remove the supernatant and resuspend the stained cell pellet with 500 μL of cold FACS buffer.
  27. Keep on ice until cell sorting.
  28. Sort mature OTII CD4^+^ T cells with a BD FACSAria^TM^ III cell sorter (Figure 2). Cells are collected in 1.5 mL Eppendorf tubes containing 100 μL of complete culture medium.
    **Figure 2.**
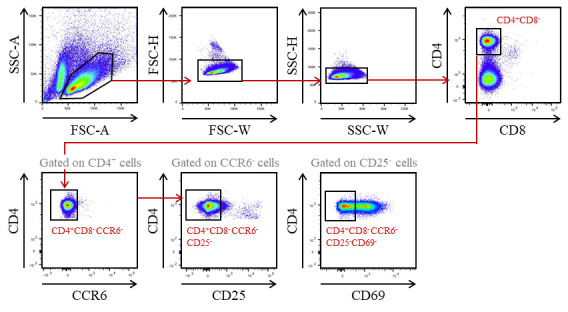
Gating strategy used to purify CD4^+^ T cells from OTIIx*Rag2*-/- mice by cell sorter after AutoMACS pre-enrichment of CD8- and CD11c- cells. Recirculating cells from the periphery into the thymus were excluded based on CCR6 expression. Mature OTII CD4^+^ T cells were gated as CD4^+^CD8-CCR6-CD25-CD69-.
- **In vitro co-culture assays**
  1. After the purification of mTEC, centrifuge the Eppendorf collection tubes at 450× *g* for 5 min at 4 °C.
  2. Remove the supernatant and carefully resuspend the cell pellet in culture medium supplemented with 5 μg/mL of OVA_323–339_ peptide.
*Note:* OVA_323–339_ *concentration can be adjusted as required.*
  3. Distribute mTEC in a plate with U bottom (100 μL/well) according to the cell concentration needed for the experimental plan.
  4. Incubate the cells in a 37 °C, 5% CO_2_ air humidified incubator for 1 h.
  5. After the purification of CD4^+^ T cells, centrifuge the Eppendorf collection tubes at 450× *g* for 5 min at 4 °C.
  6. Remove the supernatant and gently resuspend the cell pellet in complete culture medium.
  7. Add 100 μL of complete culture medium containing 10^5^ CD4^+^ T cells in each well containing OVA_323–339_-loaded mTEC.
  8. Incubate the cells in a 37 °C, 5% CO_2_ air humidified incubator for 18 h.
- **Fluorescence-activated cell sorting (FACS) analysis of OTII CD4^+^ T-cell activation after co-culture**
  1. After 18 h of co-culture, transfer the cells to a 96-well V-bottom plate for staining.
  2. Centrifuge at 450× *g* for 5 min at 4 °C.
  3. Prepare the antibody mix in cold FACS buffer for the analysis of OTII CD4^+^ T cell activation with the following concentrations:
    - Brilliant Violent 421^TM^ conjugated anti-mouse CD4 antibody: final concentration 1 μg/mL.
    - APC conjugated anti-mouse CD69 antibody: final concentration 1 μg/mL.
  4. Remove the supernatant and resuspend the cell pellet with 50 μL of antibody mix.
  5. Incubate the cells for 15 min at 4 °C.
  6. Centrifuge at 450× *g* for 5 min at 4 °C. Then, remove the supernatant and add 150 μL of cold FACS buffer.
  7. Repeat step E6.
  8. Resuspend the cells in 150 μL of cold FACS buffer.
  9. Transfer the cells to a 5 mL tube.
  10. Acquire the samples on the BD^®^ LSR II flow cytometer.

## Data analysis

**Figure 3.**
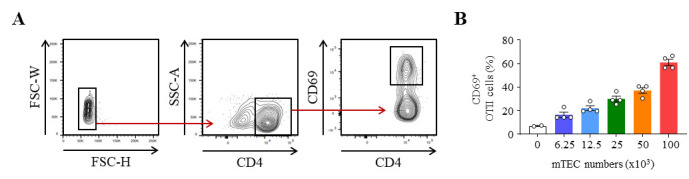
Representative results of OTII CD4^+^ T-cell activation after co-culture with OVA_323–339_-loaded medullary thymic epithelial cells (mTEC). (A) Gating strategy used to analyze the frequency of activated CD69^+^ OTII CD4^+^ T cells by flow cytometry. (B) Percentage of CD69^+^ OTII CD4^+^ T cells cultured with variable numbers of OVA_323–339_-loaded WT mTEC for 18 h.

FACS data were acquired on a BD^®^ LSR II flow cytometer using violet laser 405 nm, blue laser 488 nm, green laser 561 nm, and red laser 633 nm. FACS data were then analyzed using FlowJo software (version 10.8.1). The gating strategy used to identify the proportion of activated CD69^+^ OTII T cells after co-culture with OVA_323–339_-loaded mTEC is shown in Figure 3A. This protocol can be used with different ratios of mTEC to OTII CD4^+^ T cells to assess the antigen-presentation capacity of mTEC. As depicted in Figure 3B, the activation of OTII CD4^+^ T cells, reflected by the upregulation of CD69, gradually increases with the numbers of OVA_323–339_-loaded mTEC. The details regarding the analysis can be found in the original article, e.g., Figure 2A (Lopes et al., 2022).

## Validation of protocol

This protocol has been adapted from the previously published paper from our team (Lopes et al., 2022).

## General notes and troubleshooting

**General notes**

1. An enzymatic digestion to isolate OTII CD4^+^ T cells should be avoided since it may affect their viability and/or activation.
2. This protocol can be used in a polyclonal context.
3. This protocol can be applied to assess MHC-I antigen-presentation capacity to CD8^+^ T cells with transgenic mouse models, such as OTI mice and the cognate peptide OVA_257–264_ (SIINFEKL).
4. A viability marker can be added in steps B26, C21, and E3.
5. This protocol can be applied for a range of downstream applications, such as FACS analysis (Charaix et al., 2022; Lopes et al., 2022) or gene expression profiling (Lopes et al., 2018).
6. Each step described in the section “CD4^+^ T cell isolation” can be applied for the isolation of splenic CD4^+^ T cells and Foxp3^+^ T_reg_. This procedure was used in the publication of Charaix et al. (2022).
7. This protocol can be used to test the capacity of mTEC to generate or activate Foxp3^+^ T_reg_ (Charaix et al., 2022).

**Troubleshooting**

1. The time of digestion must be optimized to avoid over-digestion, which can lead to cell death and epitope removal.
2. To ensure an optimal pre-enrichment with the AutoMACS^®^ Pro Separator, carefully follow the manufacturer’s recommendations concerning cell concentration.
3. Clogging of the AutoMACS^®^ Pro Separator: depending on the cell density, cell suspension should be diluted and separated into two different tubes.
4. During FACS analysis: make sure to use a cytometer compatible with the fluorochromes used in the experiment, use fluorochrome combinations to avoid excessive spectral overlap, and verify compensation parameters.
5. To avoid cell death, make sure to work quickly through the protocol.
